# Evaluation of the Efficacy and Safety of Collagen Mesotherapy in the Course of Chronic Cervicocephalic Syndrome—A Retrospective Observational Study

**DOI:** 10.3390/jcm15155860

**Published:** 2026-07-27

**Authors:** Kamil Koszela, Massimo Mammucari, Michał Słupiński, Barbara Stypińska, Agnieszka Guz, Karol Myszel

**Affiliations:** 1 Department of Musculoskeletal Disorders, National Institute of Geriatrics, Rheumatology and Rehabilitation, 1 Spartańska Street, 02-637 Warsaw, Poland; 2 Polish Society of Mesotherapy, 1 Spartańska Street, 02-637 Warsaw, Poland; 3 Italian Society of Mesotherapy, ASL RM 1, 00165 Rome, Italy; 4 FiortClinic—Physiotherapy, Orthopedics, and Rehabilitation, 38 Folwarczna Street, 97-300 Piotrkow Trybunalski, Poland; 5 Department of Molecular Biology, National Institute of Geriatrics, Rheumatology, and Rehabilitation, Spartanska 1, 02-637 Warsaw, Poland; 6 Center of Hearing and Speech, 7 Mokra Street, 05-830 Kajetany, Poland; 7 Faculty of Health Sciences, University of Applied Sciences, 4 Popieluszko Street, 62-510 Konin, Poland

**Keywords:** local intradermal therapy (LIT), mesotherapy, dizziness, tinnitus, cervicocephalic syndrome

## Abstract

**Background/Objectives**: The number of patients with cervical spine pain has been increasing in recent years, and some have been diagnosed with chronic cervicocephalic syndrome (CCS). Collagen mesotherapy of the spine is increasingly being used to treat chronic pain syndromes. This retrospective observational study was conducted to evaluate the efficacy and safety of collagen mesotherapy in the course of CCS. **Methods**: The study included 31 patients (19 women and 12 men; mean age, 45.0 ± 10.1) with chronic CCS (>12 weeks) who underwent cervical spine mesotherapy using injectable collagen. Mesotherapy was performed at weekly intervals, in 5 (in 18 patients) and 6 (in 13 patients) sessions. Patients were assessed using the Visual Analogue Scale (VAS) for cervical spine pain, pain radiating to the occipital region, dizziness, and tinnitus before the start of mesotherapy, one week after its completion, and 3 months later during a follow-up visit. Changes over time were analyzed using the Friedman test for repeated measures and Cochran’s Q test for binary outcomes. **Results**: Following collagen mesotherapy, cervical pain on the VAS decreased from 7 (IQR 6–8) at baseline to 2 (IQR 1–3) after treatment and remained at 2 (IQR 1–2) after the 3-month follow-up (*p* < 0.001). The proportion of patients with occipital pain decreased from 100% to 38.7%, dizziness from 83.9% to 38.7%, and tinnitus from 19.4% to 6.5%. No adverse effects related to the therapy were observed. **Conclusions:** Collagen mesotherapy was associated with improvements in pain and accompanying symptoms in patients with chronic cervicocephalic syndrome, with no treatment-related adverse events observed. Nevertheless, further research in this area is necessary.

## 1. Introduction

The number of patients with cervical spine pain has been increasing in recent years [1]. According to data from the Global Burden of Disease, the number of cases of neck pain worldwide nearly doubled between 1990 and 2021 [2]. Typically, in addition to local pain in the cervical spine, pain radiating to the upper extremity (cervicobrachialgia) is reported, as well as pain radiating to the occipital region, which is known as cervicocephalic syndrome [3,4].

Chronic cervicocephalic syndrome (CCS) refers to a cluster of symptoms originating in the cervical spine and radiating to the head (occipital region), often accompanied by limited mobility, which may be accompanied by dizziness, nausea, tinnitus, and balance disorders. The source of the symptoms includes, among others, myofascial, degenerative, facet joint, and vertebral disc-related causes [5]. In addition to clinical symptoms, the course of cervicocephalic syndrome may be characterized by the loss of cervical lordosis [6] without other radiological changes observed upon imaging.

The anatomical proximity within the neck of nerves (particularly the sympathetic trunk, which innervates the cervical and intracranial vessels, and the somatosensory pathways responsible for conducting impulses from the proprioceptive receptors of the joints, muscles, and tendons) and the cephalic blood vessels supplying, among other structures, the inner ear, the cerebral cortex, and the cerebellum means that laryngological symptoms often occur in cervical spine pain syndrome [7,8].

One form of CCS is Barré–Liéou syndrome, described by Jean Alexandre Barré and Yong-Choen Liéou, who considered vasomotor disorders in the vertebrobasilar system a critical component. These may result in reduced blood flow in the posterior cerebral circulation and symptoms such as dizziness, headaches, and autonomic symptoms. Dizziness in cervical spine pain syndrome may also result from vertebral artery dysfunction caused by compression from bony structures or soft tissues of the neck [9,10].

These forms of dizziness, referred to as cervical vertigo, therefore, require an interdisciplinary approach involving specialists in orthopedics, otolaryngology, audiology, neurology, and physical medicine and rehabilitation. In the diagnostic process, it is essential to rule out other causes of dizziness [11].

The neural and vascular mechanisms described above are also a common cause of tinnitus reported by people with cervical spine disorders. Ischemia of the inner ear structures and the auditory nerve leads to depolarization disturbances, generating abnormal signals that are perceived by the auditory cortex as tinnitus. Rehabilitation of the cervical spine and reduction in muscle tension often lead to a reduction in tinnitus and an improvement in the patient’s well-being, as reflected in improved scores on the Tinnitus Handicap Inventory (THI) self-assessment scale [12,13].

According to the latest literature, physical therapy plays a key role in the treatment of cervicogenic headache (CGH) [14,15,16], and minimally invasive treatments such as dry needling [17] are also used. Slightly more invasive options include greater occipital nerve blocks [18,19], facet joint intra-articular injections [20], radiofrequency ablation (RFA) [21], and pulsed radiofrequency ablation (PRFA) [22], as well as botulinum toxin [23].

An increasingly common treatment option for chronic spinal pain is mesotherapy, also known as local intradermal therapy (LIT), which involves multipoint intradermal microinjections to a depth of 3–4 mm at an angle of 15–30 degrees, using specialized needles 4–12 mm long and 0.23–0.3 mm in diameter [24]. The primary goal of mesotherapy is to restore local circulation within the soft tissues. This involves relaxing tense muscles, fascia, and ligaments, thereby improving mobility and reducing pain. This effect is achieved, among other things, by stimulating skin and intradermal receptors, which activates the endogenous opioid system and increases endorphin levels [25,26].

In recent years, injectable collagen therapy, including collagen mesotherapy of the spine, has gained popularity. The efficacy and safety of collagen mesotherapy have been demonstrated in the treatment of chronic cervical myofascial pain syndrome [27,28], chronic thoracic pain syndrome [29], and chronic low back pain (LBP) [30].

It is worth noting that in recent years, the importance of mesotherapy has been emphasized in various fields of medicine, including pain management, sports medicine, rehabilitation, vascular diseases, dermatology, and esthetic medicine. International guidelines have been developed regarding the safe and evidence-based practice of mesotherapy, providing a framework for standardizing clinical practice [31,32].

The aim of this retrospective observational study is to evaluate the efficacy and safety of collagen mesotherapy of chronic cervicocephalic syndrome.

## 2. Materials and Methods

### 2.1. Study Design

This retrospective observational cohort study was based on a review of medical records and is reported in accordance with the Strengthening the Reporting of Observational Studies in Epidemiology (STROBE) Statement for cohort studies. All consecutive patients fulfilling the eligibility criteria and treated with collagen mesotherapy for chronic cervicocephalic syndrome (CCS) between 1 January 2018 and 31 December 2025 were included in the analysis. All patients were treated by the same orthopedic physician at three outpatient medical offices. Data were extracted from anonymized medical records and analyzed at the turn of 2025/2026. All patients had provided informed consent for the use of their anonymized data for research purposes. The study was approved by the Bioethics Committee at the National Institute of Geriatrics, Rheumatology, and Rehabilitation in Warsaw, Poland (27 April 2023; No. KBT-3/10/2023).

### 2.2. Inclusion and Exclusion Criteria

Inclusion criteria:

- Chronic cervicocephalic syndrome;
- A prior neurological examination ruling out causes originating in the central nervous system;
- Previous otolaryngological examinations ruling out otolaryngological causes;
- MRI of the cervical spine—no signs of spinal canal stenosis or symptomatic disc disease;
- X-ray examination of the cervical spine in anteroposterior (AP), lateral, and dynamic views (no signs of cervical spine instability);
- Doppler ultrasound of the carotid and vertebral arteries (no pathological changes).

Exclusion criteria:

- Chronic cervical spine pain syndrome of another origin;
- Inflammatory, rheumatological, or neoplastic disease;
- Previous cervical spine surgery;
- Pregnancy and/or breastfeeding;
- ENT disorders (Meniere’s disease, chronic exudative otitis media, chronic suppurative otitis media, vascular-nerve conflict, vestibulocochlear nerve tumour, otosclerosis);
- Neurological diseases (multiple sclerosis);
- Lack of patient consent to use data for publication.

### 2.3. Mesotherapy Procedure

**Figure 1:**
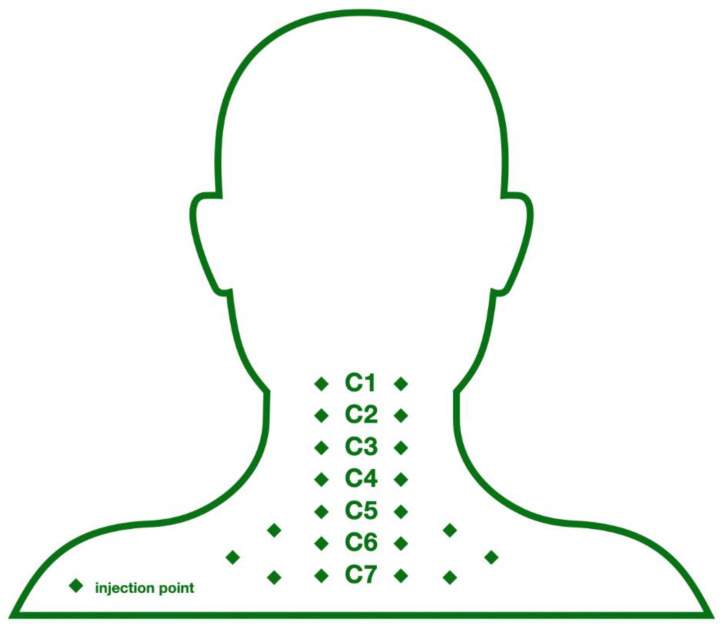
Mesotherapy protocol used in the treated patients.

Each patient underwent spinal mesotherapy following the same protocol (Figure 1), performed by the same physician using injectable type I collagen (1 ampule, 2 mL). Each patient received 20 microinjections, with 0.1 mL of the preparation administered per injection site, using a needle with a diameter of 0.3 mm and a length of 12 mm. Injections were administered bilaterally, approximately 1–1.5 cm lateral to the midline of the cervical spine at the C1–C7 levels. Additional bilateral injections were then administered approximately 3–5 cm lateral to the midline. The treatment was performed once a week, in 5 (for 18 patients) and in 6 (for 13 patients) sessions.

### 2.4. Assessment of Symptoms

During each medical visit, each patient was assessed on the Visual Analogue Scale (VAS) [33] for the presence of localized pain in the cervical spine, headache (occipital region), and the occurrence of otolaryngological symptoms (dizziness, tinnitus) before the start of mesotherapy, one week after its completion, and during the follow-up visit 3 months later.

### 2.5. Sample Size

Because this was a retrospective observational cohort study, all available patients fulfilling the eligibility criteria were included in the analysis. A sample size calculation was subsequently performed to assess whether the available cohort size was sufficient for the planned repeated-measures analysis of VAS pain scores collected at three time points: baseline, post-intervention, and follow-up. The primary global analysis was planned using the Friedman test, and if the global test was significant, pairwise post hoc comparisons were performed using the Wilcoxon signed-rank test with correction for multiple comparisons. The minimal clinically important difference was defined as a 1-point change in VAS. Based on the observed dispersion of VAS scores in the available dataset, the standard deviation of within-patient change was assumed to be 1.5 points in a moderately conservative scenario. Assuming a two-sided significance level of 0.05, 80% statistical power, and adjustment for three pairwise post hoc comparisons (adjusted α = 0.0167), a minimum of 25 evaluable patients were required. Considering a potential 15% rate of incomplete follow-up or missing data, a sample of approximately 30 patients was considered sufficient for the planned repeated-measures analysis. The final cohort consisted of 31 patients, exceeding the minimum required sample size.

### 2.6. Statistical Analysis

Statistical analyses were performed using R version 4.5.3 software (R Foundation for Statistical Computing, Vienna, Austria), with the following R packages: readxl, tidyverse, gt, rstatix, RVAideMemoire, and patchwork. Continuous variables are presented as the median and interquartile range (IQR) or mean ± standard deviation (SD), as appropriate. Categorical variables are presented as counts and percentages. Changes in pain intensity (VAS) across the three assessment time points were evaluated using the Friedman test for repeated measures. Changes in binary outcomes (occipital pain, dizziness, and tinnitus) over time were assessed using Cochran’s Q test. When significant, pairwise post hoc comparisons were performed using the Wilcoxon signed-rank test for VAS scores and exact McNemar tests for binary outcomes, with Bonferroni correction for multiple comparisons. Statistical significance was set at *p* < 0.05.

## 3. Results

This retrospective observational study included 31 patients: 19 women and 12 men aged 30 to 64 years (Table 1) with CCS.

**Figure 2:**
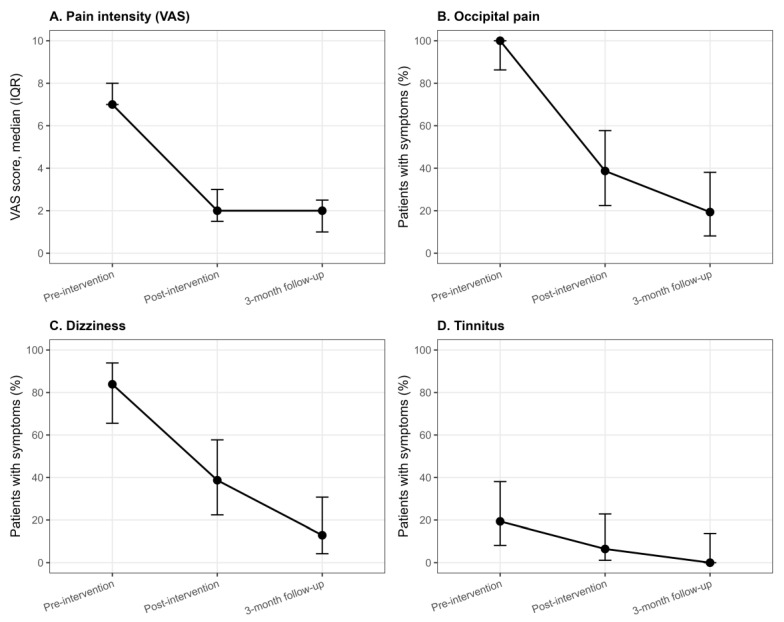
Changes in pain intensity and associated symptoms over time following intervention. Figure (**A**) presents pain intensity measured using the Visual Analogue Scale (VAS). Data are shown as median and interquartile range (IQR). Figure (**B**–**D**) present the proportion of patients reporting occipital pain, dizziness, and tinnitus, respectively. Error bars represent 95% confidence intervals for proportions. Assessments were performed at baseline (pre-intervention), immediately after treatment (post-intervention), and at the 3-month follow-up.

Collagen mesotherapy of the cervical spine in patients with CCS resulted in a clinically significant reduction in pain on the VAS after completion of therapy and following a 3-month follow-up during a follow-up visit (Table 2, Figure 2A, Supplementary Table S1). Pairwise post hoc analyses confirmed significant reductions in VAS scores between all assessment time points (all Bonferroni-adjusted *p* < 0.05).

In addition, a reduction in symptoms of pain radiating to the occipital region, dizziness, and tinnitus was observed following collagen mesotherapy of the cervical spine (Table 2, Figure 2B–D). Pairwise post hoc analyses demonstrated significant reductions in occipital pain between baseline and both post-intervention assessments, whereas no significant difference was observed between the post-intervention and 3-month follow-up assessments. For dizziness, significant reductions were observed between baseline and both post-intervention assessments, whereas the difference between the post-intervention and 3-month follow-up assessments was not significant after Bonferroni correction. For tinnitus, none of the pairwise comparisons remained statistically significant after Bonferroni correction, although the overall Cochran’s Q test was significant. Detailed post hoc results are presented in Supplementary Table S2.

During treatment and the follow-up period, no adverse effects related to collagen mesotherapy were observed, and no allergic reactions were noted during treatment. Nevertheless, patients reported localized pain in the cervical region associated with the injections.

## 4. Discussion

This is the first retrospective observational study evaluating the efficacy and safety of collagen mesotherapy in patients with CCS.

Patients with cervical spine pain accompanied by headaches (in the occipital region), dizziness, or tinnitus are relatively challenging. The first step is to rule out neurological, otolaryngological, and sometimes even ophthalmological causes, and it is often necessary to perform specialized imaging studies such as MRI and/or CT scans of the head, as well as Doppler ultrasound of the carotid and vertebral arteries. Diagnostic difficulties stem from the nonspecific clinical presentation, the lack of definitive tests to confirm the diagnosis, and the complex anatomy of the neck. The literature lacks clear diagnostic criteria, which makes it difficult to compare studies and clinical practice. Furthermore, the diagnosis of CCS is often made by ruling out other pathologies, as evidenced by the publication by Reiley et al. [34].

To date, studies have demonstrated the efficacy and safety of collagen mesotherapy in the management of chronic cervical myofascial pain syndrome. Similarly to this study, patients were assessed using the VAS before the start of therapy, after its completion, and during a 3-month follow-up visit. Similar studies have been conducted on the thoracic and lumbosacral spine. Moreover, in these studies, apart from pain reduction, the following were observed: functional improvement and reduction in the intake of painkillers [27,28,29,30].

According to the latest reports, combined interventions, such as spinal manipulation combined with dry needling and muscle energy techniques or soft tissue techniques, or dry needling combined with exercises, appear to be the most effective interventions for reducing the intensity and/or frequency of short-term headaches of cervical origin [35].

Following a randomized study, Mousavi-Khatir et al. emphasized that dry needling is an effective, minimally invasive treatment for CCS. It targets myofascial trigger points in the neck, upper back, and suboccipital muscles to reduce referred pain, alleviate dizziness, and relieve cervicogenic headaches [36]. Dry needling, like mesotherapy, stimulates receptors in the skin and subcutaneous tissue, triggering a series of repair mechanisms. It helps normalize myofascial tension, improves local circulation, and stimulates tissue regeneration [37,38]. In mesotherapy, in addition to mechanical irritation from the needle, medications, medical devices, and combinations administered may also cause therapeutic effect. In collagen mesotherapy, additional repair mechanisms are activated [24,25]. Based on research conducted on human gluteal tenocytes, it was observed that injectable collagen induces fibroblast proliferation and migration to the site of injury, and stimulates the synthesis, secretion, and maturation of type I collagen (COL-I). Furthermore, it inhibits the synthesis of matrix metalloproteinase type I (MMP-1), which degrades COL [39,40]. Nevertheless, further research is necessary in the context of spinal pathology.

It is worth noting the systematic review published by Goyal et al. [41], which concluded that occipital nerve blocks, facet joint injections, deep cervical plexus blocks, and epidural injections in the neck region may be reasonable options for refractory cases of cervicogenic headache (CeH). Nevertheless, these procedures are significantly more invasive and require specialized equipment and extensive physician experience compared to cervical spine mesotherapy.

In recent years, numerous publications have appeared on the efficacy and safety of mesotherapy in managing chronic cervical spine pain syndrome using various medications and combinations thereof. Paolucci et al. [42] demonstrated the efficacy of trigger point mesotherapy in the treatment of chronic neck pain. In this study, a significant decrease in symptoms on the VAS was observed after using lignocaine mesotherapy compared to dry mesotherapy. Scaturro et al. [43] demonstrated the efficacy of mesotherapy combined with exercises for neck pain associated with fibromyalgia. This study showed a decrease in pain and significant statistical improvements in functional capacity. By contrast, Ranieri et al. [44] demonstrated the efficacy of mesotherapy in bilateral cervicobrachial pain. In this study, a decrease in pain on the VAS, improvement in neck mobility and improved stiffness value were observed. No adverse effects related to the therapy were reported in the analyzed publications, nor were any reported in this study.

Multifactorial rehabilitation for cervical spine pain syndrome (including mesotherapy), by reducing excessive muscle tension, alleviating local inflammation, and improving blood flow, can also effectively help alleviate or eliminate otolaryngological symptoms such as dizziness or tinnitus. Reducing excessive sympathetic nervous system activation—including through psychogenic mechanisms (improving the patient’s well-being and quality of life)—can significantly reduce the number of symptoms reported by the patient [45,46,47].

A meta-analysis published by Jin et al. in 2021 [48] demonstrated the efficacy and safety of manual therapy in CCS treatment, with lower scores on the VAS, Dizziness Handicap Inventory (DHI), and Neck Disability Index (NDI), as well as improved cervical spine mobility.

Due to the minimally invasive nature and safety of the therapy, the role of mesotherapy in pain medicine is growing. An important aspect is the published international guidelines, which define the indications and methods of applying this therapy in various pathologies [31,32].

A major limitation of this study is its retrospective nature and the absence of a comparison or control group. Its small sample size is also a limitation; however, the limited sample size was due to the restrictive inclusion and exclusion criteria. It should be noted that only six participants presented with tinnitus at baseline; therefore, the related findings should be interpreted with caution and require confirmation in adequately powered studies specifically designed to evaluate tinnitus outcomes. Furthermore, it cannot be ruled out that during the 3-month follow-up period, patients may have taken analgesics, anti-inflammatory drugs, or muscle relaxants as needed or undergone physical therapy, which could have influenced the results. However, patients who reported using the above-mentioned therapies were excluded from the study. Eighteen patients underwent 5 mesotherapy sessions and 13 patients underwent 6 mesotherapy sessions at weekly intervals, which may have influenced the results. Furthermore, the assessment of pain radiating to the occipital region, dizziness, and tinnitus was subjective rather than based on specialized scales.

Further randomized (double-blind) studies are needed to compare various injection techniques (e.g., multipoint intramuscular injections), various medications (including drug combinations), and other therapeutic techniques in CCS management. Additionally, multi-centre studies conducted by different physicians could reduce the risk of bias. Furthermore, it would be worthwhile to evaluate X-rays of the cervical spine after treatment and assess sagittal balance parameters.

## 5. Conclusions

In this retrospective observational cohort, collagen mesotherapy was associated with substantial improvements in pain and accompanying symptoms in patients with chronic cervicocephalic syndrome, and no treatment-related adverse events were observed. Although these findings suggest that collagen mesotherapy may represent a promising therapeutic option, the retrospective design and lack of a control group preclude definitive conclusions regarding its efficacy.

## Figures and Tables

**Table 1:** Baseline demographic characteristics.

	Overall (*n* = 31)
Age, years	45.0 ± 10.1
Female	19 (61.3%)
Male	12 (38.7%)

Data are presented as mean ± standard deviation (SD) or *n* (%).

**Table 2:** Clinical outcomes over time.

Outcome	Pre-Intervention	Post-Intervention	3-Month Follow-Up	*p*-Value
VAS score	7.0 (7.0–8.0)	2.0 (1.5–3.0)	2.0 (1.0–2.5)	<0.001
Occipital pain, *n* (%)	31 (100.0%)	12 (38.7%)	6 (19.4%)	<0.001
Dizziness, *n* (%)	26 (83.9%)	12 (38.7%)	4 (12.9%)	<0.001
Tinnitus, *n* (%)	6 (19.4%)	2 (6.5%)	0 (0.0%)	0.009

VAS values are presented as median (IQR). Occipital pain, dizziness, and tinnitus are presented as *n* (%). *p*-values were calculated using the Friedman test for VAS and Cochran’s Q test for binary outcomes.

## Data Availability

The data are available from the corresponding author upon request.

## Supplementary Materials

The following supporting information can be downloaded at: https://www.mdpi.com/article/10.3390/jcm15155860/s1, Supplementary Table S1. Changes in VAS relative to baseline; Supplementary Table S2. Pairwise post-hoc comparisons.
