# Supplementary material for: Evaluation of the Efficacy and Safety of Collagen Mesotherapy in the Course of Chronic Cervicocephalic Syndrome—A Retrospective Observational Study
Source: J Clin Med. 2026 Jul 27;15(15):5860. doi: 10.3390/jcm15155860 (PMC13466833; doi:10.3390/jcm15155860)
Supplement: Supplementary file 1 [file jcm-15-05860-s001.zip › jcm-4440241-supplementary.pdf]

Supplementary Table S1. Changes in VAS relative to baseline

Comparison	Median Δ (IQR)	95% CI	Wilcoxon p
Post-intervention − Baseline	-5.0 (-6.0–-4.0)	-6.0 to -4.0	<0.001
3-month follow-up − Baseline	-5.0 (-6.0–-5.0)	-6.0 to -5.0	<0.001

Values represent paired changes in VAS relative to baseline. Median differences with interquartile ranges (IQR), bootstrap 95% confidence intervals (5,000 resamples), and paired Wilcoxon signed-rank test p-values are presented.

Supplementary Table S2. Pairwise post-hoc comparisons

Outcome	Comparison	Test	Discordant pairs	Raw p-value	Bonferroni-adjusted p-value
VAS score	Pre-intervention vs Post-intervention	Wilcoxon signed-rank test	NA	<0.001	<0.001
VAS score	Pre-intervention vs 3-month follow-up	Wilcoxon signed-rank test	NA	<0.001	<0.001
VAS score	Post-intervention vs 3-month follow-up	Wilcoxon signed-rank test	NA	0.009	0.027
Occipital pain	Pre-intervention vs Post-intervention	Exact McNemar test	19	<0.001	<0.001
Occipital pain	Pre-intervention vs 3-month follow-up	Exact McNemar test	25	<0.001	<0.001
Occipital pain	Post-intervention vs 3-month follow-up	Exact McNemar test	12	0.146	0.438
Dizziness	Pre-intervention vs Post-intervention	Exact McNemar test	14	<0.001	<0.001
Dizziness	Pre-intervention vs 3-month follow-up	Exact McNemar test	22	<0.001	<0.001

Supplementary Table S2. Pairwise post-hoc comparisons

Outcome	Comparison	Test	Discordant pairs	Raw p-value	Bonferroni-adjusted p-value
Dizziness	Post-intervention vs 3-month follow-up	Exact McNemar test	10	0.021	0.064
Tinnitus	Pre-intervention vs Post-intervention	Exact McNemar test	4	0.125	0.375
Tinnitus	Pre-intervention vs 3-month follow-up	Exact McNemar test	6	0.031	0.094
Tinnitus	Post-intervention vs 3-month follow-up	Exact McNemar test	2	0.500	1.000

Pairwise comparisons were performed using the Wilcoxon signed-rank test for VAS and exact McNemar tests for binary outcomes. P-values were adjusted using the Bonferroni correction within each outcome.
